# Supplementary material for: Molecular mechanisms of coronary artery disease risk at the PDGFD locus
Source: Nat Commun. 2023 Feb 15;14:847. doi: 10.1038/s41467-023-36518-9 (PMC9932166; doi:10.1038/s41467-023-36518-9)
Supplement: Supplementary file 1 — Supplementary Information [file 41467_2023_36518_MOESM1_ESM.pdf]

Supplemental Materials - Kim et al., “Molecular mechanisms of coronary artery disease risk at the PDGFD locus”

Suppl. Fig. 1

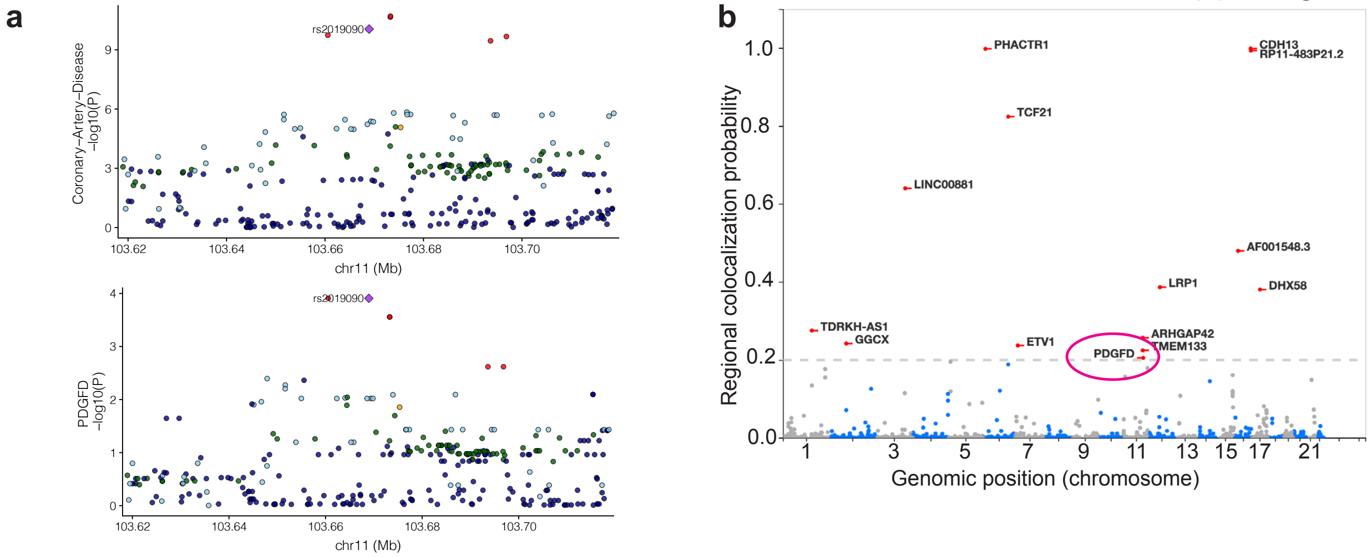

c

Pi-1- ATAAATTTAGGCTAGACCAA - specificity score 62, efficiency score 58,
Location: second base at 5’ end overlaps with rs2019090

Pi-2 - CCAAAGGGACTGCCAGACTG - specificty score 57, efficiency score 67,
Location: 14bp downstream of rs2019090

Pi-3 - TATAAAGGGTCACTATCTGG - specificity score 76, efficiency score 63,
Location: 72bp upstream of rs2019090

Suppl. Figure 1. Functional variant rs2019090 is associated with CAD risk and PDGFD expression, and upstream regulator FOXC1/C2 is predicted to also be CAD associated. (a) Correlation of rs2019090 eQTL activity toward PDGFD and CAD GWAS association. (b) Colocalization of PDGFD CAD GWAS association at 11q23.2 and regulation of PDGFD expression was performed with the enloc genome-wide co-localization analysis algorithm. Regional colocalization probability >2 was considered significant. (c) Sequence and targeted location for guide RNAs targeted to rs2019090.

Suppl. Fig. 2

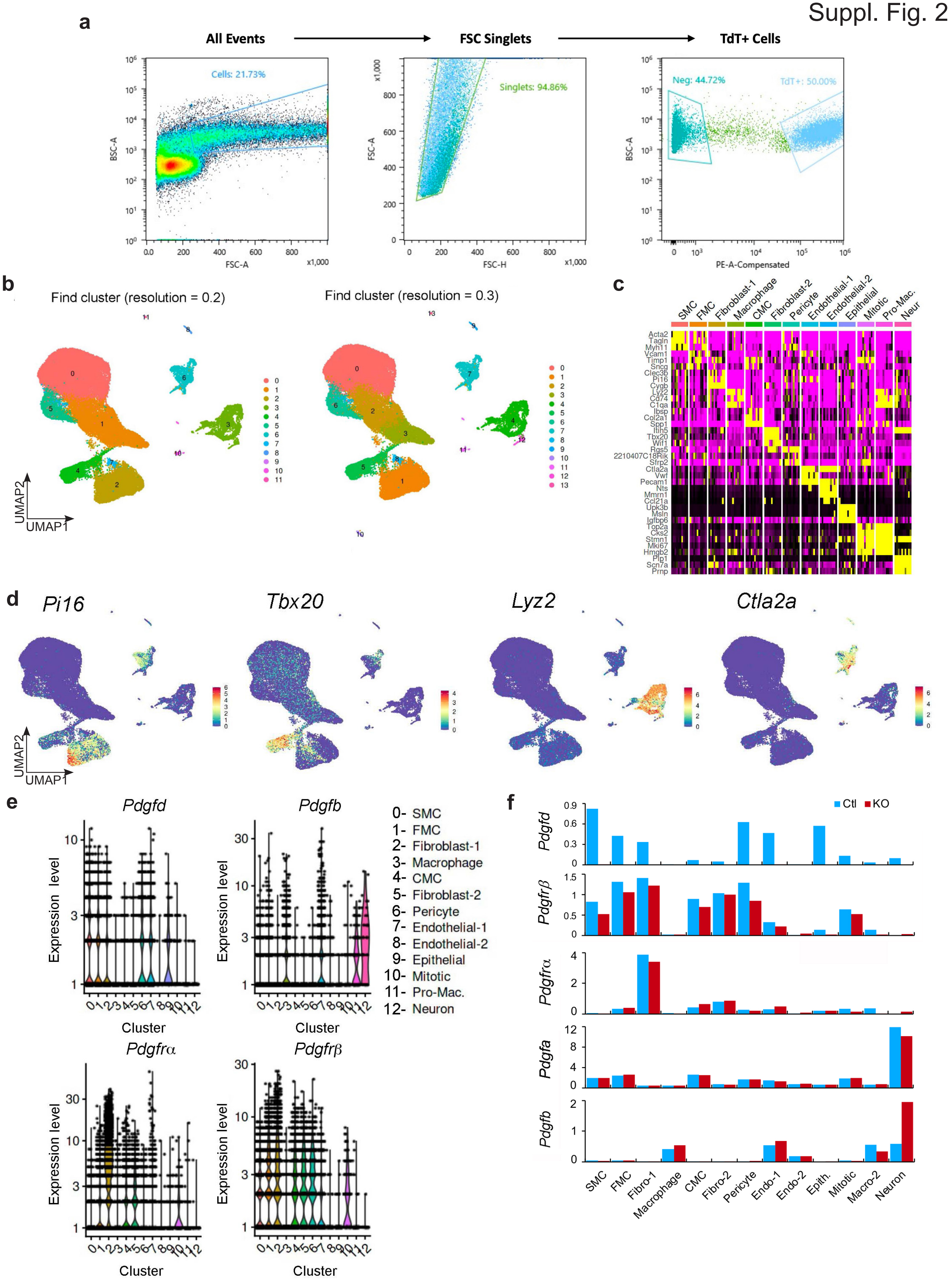
a
All Events
FSC Singlets
TdT+ Cells
Cells: 21.73%
BSC-A
FSC-A
x1,000
Singlets: 94.86%
FSC-A
FSC-H
Neg: 44.72%
TdT+: 50.00%
PE-A-Compensated
b
Find cluster (resolution = 0.2)
Find cluster (resolution = 0.3)
UMAP2
UMAP1
c
SMC
FMC
Fibroblast-1
Macrophage
CMC
Fibroblast-2
Pericyte
Endothelial-1
Endothelial-2
Epithelial
Mitotic
Pro-Mac.
Neur
Acta2
Tagln
Myh11
Vcam1
Timp1
Sncg
Clec3b
Pi16
Cygb
Lyz2
Cd74
C1qa
Ibsp
Col2a1
Spp1
Itln5
Tbx20
Wif1
Rgs5
2210407C18Rik
Sfrp2
Ctla2a
Vwf
Pecam1
Nts
Mmrn1
Ccl21a
Upk3b
Msln
Igfbp6
Top2a
Cks2
Stmn1
Mki67
Hmgb2
Plp1
Scn7a
Prnp
d
Pi16
Tbx20
Lyz2
Ctla2a
UMAP2
UMAP1
e
Pdgfd
Pdgfb
Expression level
Cluster
0- SMC
1- FMC
2- Fibroblast-1
3- Macrophage
4- CMC
5- Fibroblast-2
6- Pericyte
7- Endothelial-1
8- Endothelial-2
9- Epithelial
10- Mitotic
11- Pro-Mac.
12- Neuron
Pdgfrα
Pdgfrβ
Expression level
Cluster
f
Ctl
KO
Pdgfd
Pdgfrβ
Pdgfrα
Pdgfa
Pdgfb
SMC
FMC
Fibro-1
Macrophage
CMC
Fibro-2
Pericyte
Endo-1
Endo-2
Epith.
Mitotic
Macro-2
Neuron

Suppl. Figure 2. Single cell RNA sequencing data analyses. (a) Representative FACS strategy to analyse Tdt positive and negative populations in dissected mouse aortic roots. (b) UMAP displaying unbiased Seurat clustering of the total scRNAseq dataset at a lower (0.2) and higher resolution (0.3) than the optimal chosen resolution shown in Fig. 3B. (c) Heat map displaying top three genes defining each cell cluster identity. (d) Feature plots showing expression of unique cluster markers not shown in Fig. 3C: Pi16, fibroblast-1; Tbx20, fibroblast-2; Lyz2, macrophage; Ctla2, endothelial-1. (e) Violin plots visualizing single-cell expression distributions in each cluster for Pdgfd, Pdgfb, Pdgfra, and Pdgfrb. (f) Comparison of average expression values in individual clusters between Ctl and KO for Pdgfd, Pdgfrb, Pdgfra, Pdgfa, and Pdgfb.

Suppl. Fig. 3

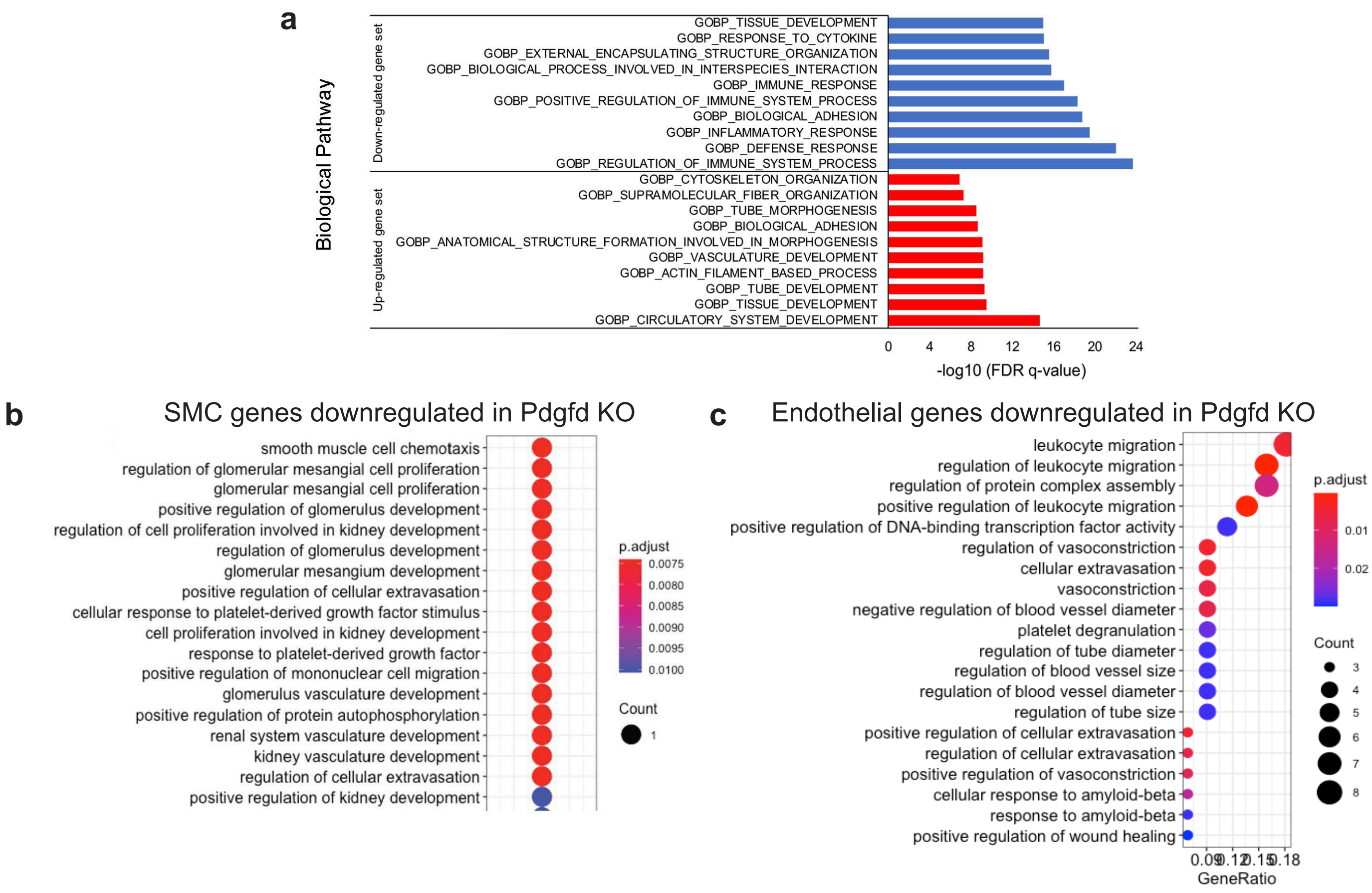

Suppl. Figure 3. Pathways enriched with Pdgfd regulated genes. (a) Bar plots of biological pathways enriched in down-regulated DEGs identified across all clusters when KO animals were compared to Ctl animals. Enrichment pathways were predicted by MsigDB database v7.5.1. and gene set enrichment analysis (GSEA), with permutation tests to determine adjusted p-value. (b) Biological processes enriched with down-regulated DEGs from Pdgfd KO compared to Ctl mice identified for cells in the SMC cluster and (c) Endo-1 cluster as determined by clusterProfiler and the GSEA statistical tool.

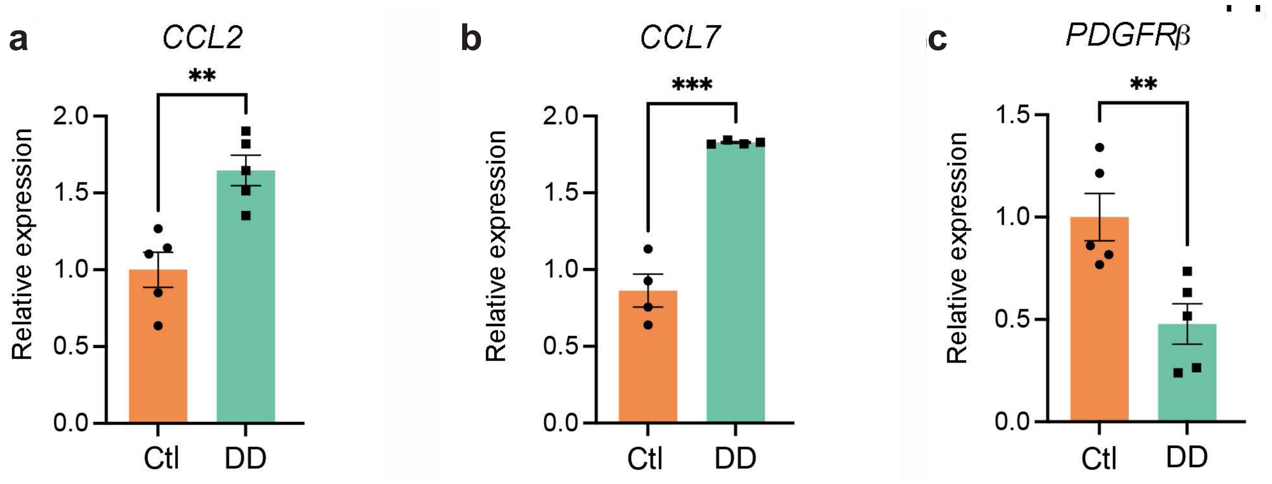

Suppl. Figure 4. In vitro qPCR validation of fibroblast chemokine response to PDGFDD. qPCR showed that (a) CCL2, p=0.0027 and (b) CCL7 expression, p=0.0001 was increased, and (c) PDGFRB expression, p=0.0089 decreased in IMR90 human fibroblasts when treated with PDGFDD (50 ng/ml) for 24 hr after 24 hr-serum starvation. Values represent mean ± s.e.m of three technical replicates, expressed as fold change relative to control with p-values obtained with two-sided unpaired t-test. Source data are provided as a Source Data file.

Suppl. Fig. 5

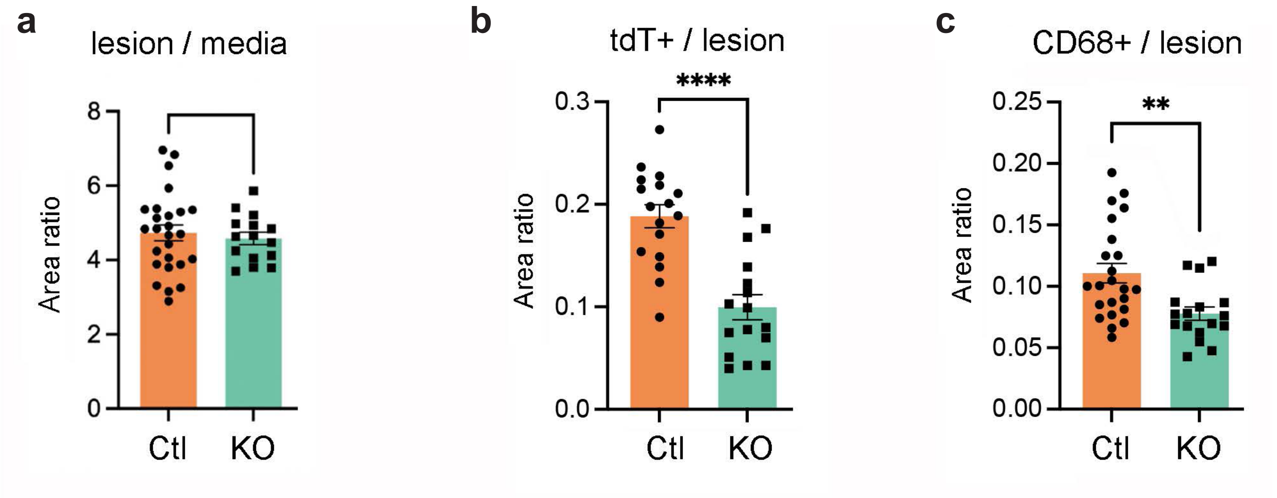

Suppl. Figure 5. Quantification of relative lesion area features. (a) Quantification of relative lesion area normalized to medial area, n = 26 control and 15 KO mouse sections (b) quantification of relative tdT positive area in lesion, n = 17 control and 16 KO mouse sections, $p<0.0001$ (c) quantification of Cd68 positive area normalized to lesion area n = 23 control and 17 KO mouse sections, $p=0.0032$. Each dot represents quantification from identical level sections from individual animals. Data expressed as mean ± s.e.m with p-values using a two-sided unpaired t-test.

Suppl. Fig. 6

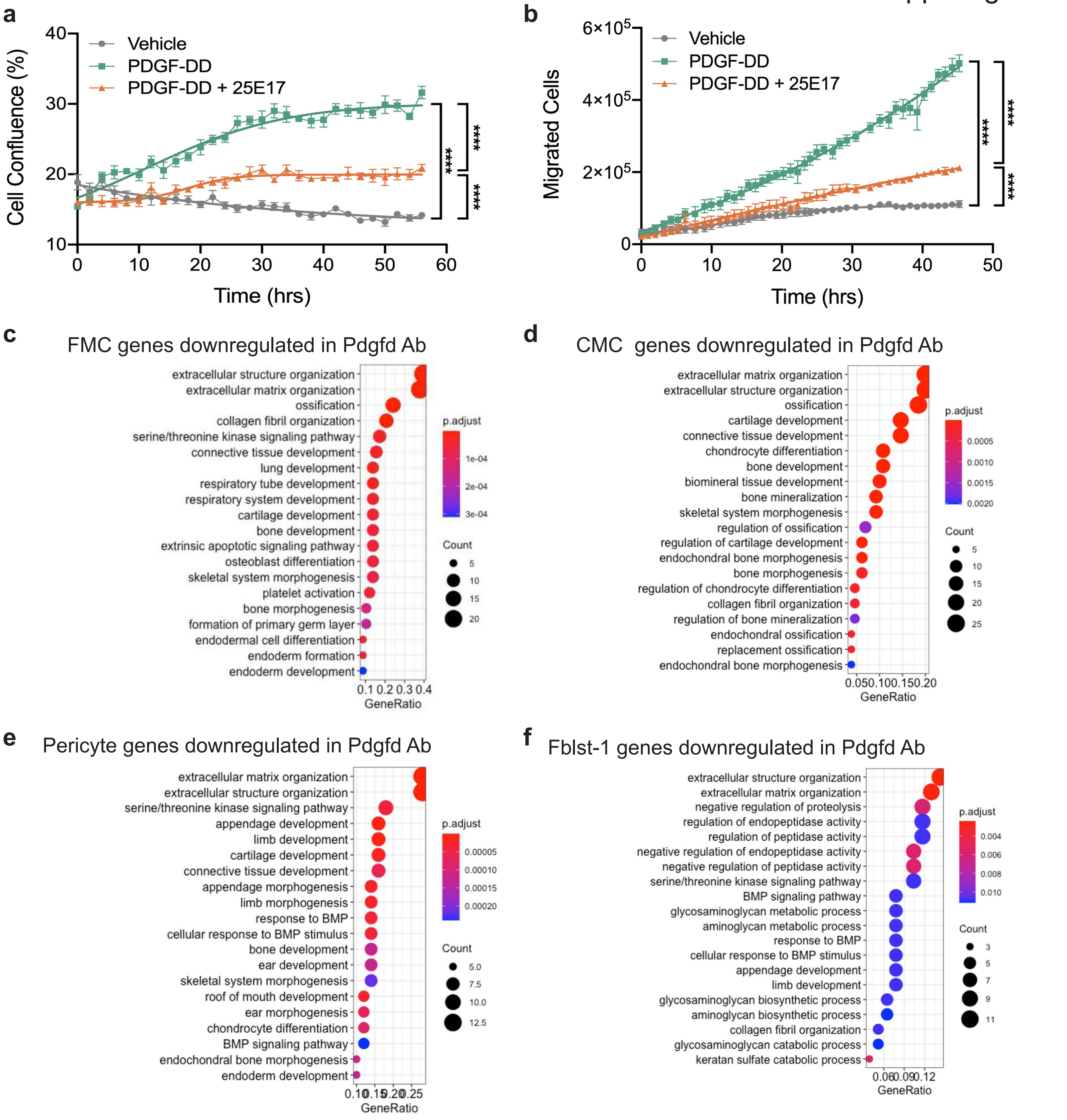

Suppl. Figure 6. Pdgfd antibody blocking study. (a) Human aortic smooth muscle cell (HASMC) proliferative response to PDGFDD and blockade with Pdgfd antibody, n = 4 biologically independent samples for vehicle, n = 6 biologically independent samples for PDGF-DD, n = 5 biologically independent samples for PDGF-DD+25E17, p=3.050E-06, p=7.706E-08, p=0.0002. (b) HASMC migration in response to PDGFDD and blockade with Pdgfd antibody 25E17, n = 5 biologically independent samples, p=3.288E-06, p=2.140E-06, p=0.0002. Values represent mean ± s.e.m. PDGFDD was used at 17.9 nM and Pdgfd antibody at 35.7 nM. Two-tailed Student t-test with two sample equal variance was used to determine p-value. All source data are provided as Source Data file. (c-f) Pathway analyses for DEGs identified after 16 weeks high fat diet and 13 weeks antibody treatment. Graphs depict biological process identified for down-regulated DEGs for (c) FMC, (d) CMC, (e) Pericytes, and (f) Fblst-1 cells as determined by clusterProfiler. Gene set enrichment analysis (GSEA) was used to determine statistical significant level of enrichment.

Supplemental Data Tables

Supplemental Data 1. Top 30 mouse cell cluster markers distinguishing each cluster (reference cluster) from the remaining clusters.

Supplemental Data 2. Differentially regulated genes per cluster in Pdgfd knockout compared to widltype animals.

Supplemental Data 3. Differentially regulated genes per cluster in Pdgfd antibody treated compared to wild-type animals.
