# Supplementary material for: Molecular mechanisms of coronary artery disease risk at the PDGFD locus
Source: Nat Commun. 2023 Feb 15;14:847. doi: 10.1038/s41467-023-36518-9 (PMC9932166; doi:10.1038/s41467-023-36518-9)
Supplement: Supplementary file 3 — Description of Additional Supplementary Files [file 41467_2023_36518_MOESM3_ESM.pdf]

Description of Additional Supplementary Files:

Supplementary Data 1. Top 30 mouse cell cluster markers distinguishing each cluster (reference cluster) from the remaining clusters.

Supplementary Data 2. Differentially regulated genes per cluster in Pdgfd knockout compared to wildtype animals.

Supplementary Data 3. Differentially regulated genes per cluster in Pdgfd antibody treated compared to widltype animals.
