# Supplementary material for: Molecular mechanisms of coronary artery disease risk at the PDGFD locus
Source: Nat Commun. 2023 Feb 15;14:847. doi: 10.1038/s41467-023-36518-9 (PMC9932166; doi:10.1038/s41467-023-36518-9)
Supplement: Supplementary file 7 — Reporting Summary [file 41467_2023_36518_MOESM7_ESM.pdf]

nature portfolio

Corresponding author(s): Thomas Quertermous

Last updated by author(s): Jan 1, 2023

Reporting Summary

Nature Portfolio wishes to improve the reproducibility of the work that we publish. This form provides structure for consistency and transparency in reporting. For further information on Nature Portfolio policies, see our Editorial Policies and the Editorial Policy Checklist.

Statistics

For all statistical analyses, confirm that the following items are present in the figure legend, table legend, main text, or Methods section.

n/a	Confirmed	
☐	☒	The exact sample size (*n*) for each experimental group/condition, given as a discrete number and unit of measurement
☐	☒	A statement on whether measurements were taken from distinct samples or whether the same sample was measured repeatedly
☐	☒	The statistical test(s) used AND whether they are one- or two-sided<br>*Only common tests should be described solely by name; describe more complex techniques in the Methods section.*
☐	☒	A description of all covariates tested
☐	☒	A description of any assumptions or corrections, such as tests of normality and adjustment for multiple comparisons
☐	☒	A full description of the statistical parameters including central tendency (e.g. means) or other basic estimates (e.g. regression coefficient) AND variation (e.g. standard deviation) or associated estimates of uncertainty (e.g. confidence intervals)
☐	☒	For null hypothesis testing, the test statistic (e.g. *F*, *t*, *r*) with confidence intervals, effect sizes, degrees of freedom and *P* value noted<br>*Give P values as exact values whenever suitable.*
☐	☐	For Bayesian analysis, information on the choice of priors and Markov chain Monte Carlo settings
☐	☐	For hierarchical and complex designs, identification of the appropriate level for tests and full reporting of outcomes
☐	☐	Estimates of effect sizes (e.g. Cohen's *d*, Pearson's *r*), indicating how they were calculated

Our web collection on statistics for biologists contains articles on many of the points above.

Software and code

Policy information about availability of computer code

Data collection	Colocalization was performed with the enloc algorithm v1.0, single cell fastq files mapped with CellRanger (7.1.0). Fluorescence cytometry, FACS software for Sony machine LE-SH800 Series software v1.7, and FlowJo version 10.6.2, and image acquisition with Leica Application Suite X software (LAS X 5.1.0).
Data analysis	Seurat (v4.0) was used for clustering and differential gene expression (FindMarker) analysis, and heatmap generation (DoHeatmap). Graphing and statistical analysis was provided by GraphPad PRISM (v8), image analysis was performed with NIH Image (v.53t 24), FACS software for Sony machine LE-SH800 Series software v1.7, and FlowJo version 10.6.2 (FlowJo; for flow cytometry data). Realtime qPCR was provided with ViiA7 Real-Time PCR system (Applied Biosystems, Foster City, CA) Software: ViiA 7 Software version 1.2.1.

For manuscripts utilizing custom algorithms or software that are central to the research but not yet described in published literature, software must be made available to editors and reviewers. We strongly encourage code deposition in a community repository (e.g. GitHub). See the Nature Portfolio guidelines for submitting code & software for further information.

Data

Policy information about availability of data

All manuscripts must include a data availability statement. This statement should provide the following information, where applicable:

- Accession codes, unique identifiers, or web links for publicly available datasets
- A description of any restrictions on data availability
- For clinical datasets or third party data, please ensure that the statement adheres to our policy

Primary data generated through this project has been uploaded to the Gene Expression Omnibus at the NIH, project number PRJNA882087, file access at GSE214423, and these data have been released for public access. Data from the following repositories was employed: NHGRI-EBI GWAS Catalog (V1.0.2) (https://

www.ebi.ac.uk/gwas/), mouse genome mm10 (https://www.ncbi.nlm.nih.gov/grc/mouse), GTEx v8 (https://gtexportal.org/home/), JASPAR transcription factor motif database (JASPAR motif database) (https://jaspar.genereg.net/).

Field-specific reporting

Please select the one below that is the best fit for your research. If you are not sure, read the appropriate sections before making your selection.

[x] Life sciences [ ] Behavioural & social sciences [ ] Ecological, evolutionary & environmental sciences

For a reference copy of the document with all sections, see nature.com/documents/nr-reporting-summary-flat.pdf

Life sciences study design

All studies must disclose on these points even when the disclosure is negative.

Sample size	Sample size was determined based on extensive experience with similar experiments in our laboratory (PMID: 36246779, PMID: 34990206, PMID: 32441123). Exact sample size for each experiment is indicated in the figure legends for each panel. These sample sizes were sufficient because they provided statistically significant results that were cross-validated with other modalities, for instance single cell RNA sequencing data and histology data for cell number were used for cross-validation.
Data exclusions	The number of genes, number of unique molecular identifiers and the percentage of mitochondrial genes were examined to identify outliers. As an unusually high number of genes can result from a 'doublet' event, in which two different cell types are captured together with the same barcoded bead, cells with >6000 genes were discarded. Cells containing >7.5% mitochondrial genes were presumed to be of poor quality and were also discarded. There were no other exclusions.
Replication	All attempts of replication were successful. All experiments were performed at least three times, with consistent results. Experiments were conducted with at least three technical replicates, exact number of biological replicates can be found in the figure legends.
Randomization	For single cell RNAseq of knockout animals, there was no randomization possible. All animals used for the antibody blocking were selected and grouped at random.
Blinding	Workers were blinded to sample genotype identity for the Pdgfd knockout lesion analysis, and for antibody blockade lesion analyses.

Reporting for specific materials, systems and methods

We require information from authors about some types of materials, experimental systems and methods used in many studies. Here, indicate whether each material, system or method listed is relevant to your study. If you are not sure if a list item applies to your research, read the appropriate section before selecting a response.

Materials & experimental systems

n/a	Involved in the study	
[ ]	[x]	Antibodies
[ ]	[x]	Eukaryotic cell lines
[ ]	[ ]	Palaeontology and archaeology
[ ]	[x]	Animals and other organisms
[ ]	[ ]	Human research participants
[ ]	[ ]	Clinical data
[ ]	[ ]	Dual use research of concern

Methods

n/a	Involved in the study	
[ ]	[ ]	ChIP-seq
[ ]	[x]	Flow cytometry
[ ]	[ ]	MRI-based neuroimaging

Antibodies

Antibodies used	Lesion histology cell areas were determined with Cnn1 rabbit monoclonal primary antibody at 1:400 dilution (TA327614; Origene), and CD68 rabbit polyclonal antibody at 1:300 dilution (ab125212; Abcam). Lots were not recorded. Blocking antibody Pdgfd 25E17, PDGFD-ab was employed in in vitro blocking and in vivo blocking experiments. An IgG1 isotype control antibody was custom developed to serve as a negative control for the Pdgfd blocking antibody.
Validation	Testing was performed with slides containing cells of known origin and known CD68 or Cnn1 staining. Pdgfd antibody 25E17 experimental validation is presented in Supplemental Figure 7. The IgG1 control antibody was identified at random and evaluated through studies reported here, results are shown in Supplemental Figure 7.

Eukaryotic cell lines

Policy information about cell lines

Cell line source(s)

Primary HCASMC were purchased from Cell Applications, Inc (San Diego, CA) and Lonza BioScience and were cultured in complete smooth muscle basal media (Lonza, #CC-3182) according to the manufacturer's instructions. All experiments were performed with HCASMC between passages 5–8. Rat aortic smooth muscle cells (A7r5) and human embryonic kidney 293 cells (HEK293) were purchased from ATCC and cultured in Dulbecco's Modified Eagle Medium (DMEM) high glucose (Fisher Scientific, #MT10013CV) with 10% FBS at 37 ºC and 5% CO2. A7r5 at passage 6-18 were used for experiments. IMR90 fetal lung fibroblasts at passage 7 were cultured in FGM-2 lung fibroblast basal media (Lonza, #CC-3131) according to the manufacturer's instructions.

Authentication

Smooth muscle cells are not cell lines, but primary cultured cells sustained in culture, with no available genetic information, so authentication regarding their authenticity is not a concerns. These cells were authenticated to be human smooth muscle cells by gene expression and morphology and phenotypic changes with stimulations. Other cell lines were validated by RT-PCR for a panel of expressed genes.

Mycoplasma contamination

Testing was regularly performed in all cell populations studied.

Commonly misidentified lines
(See ICLAC register)

No commonly misidentified lines were used in these studies.

Palaeontology and Archaeology

Specimen provenance

Provide provenance information for specimens and describe permits that were obtained for the work (including the name of the issuing authority, the date of issue, and any identifying information). Permits should encompass collection and, where applicable, export.

Specimen deposition

Indicate where the specimens have been deposited to permit free access by other researchers.

Dating methods

If new dates are provided, describe how they were obtained (e.g. collection, storage, sample pretreatment and measurement), where they were obtained (i.e. lab name), the calibration program and the protocol for quality assurance OR state that no new dates are provided.

☐ Tick this box to confirm that the raw and calibrated dates are available in the paper or in Supplementary Information.

Ethics oversight

Identify the organization(s) that approved or provided guidance on the study protocol, OR state that no ethical approval or guidance was required and explain why not.

Note that full information on the approval of the study protocol must also be provided in the manuscript.

Animals and other organisms

Policy information about studies involving animals; ARRIVE guidelines recommended for reporting animal research

Laboratory animals

We used a Pdgfd constitutive null line (Pdgfdtm1Ueks) on the C57Bl/6 background and was transferred to us by Ulf Eriksson at the Karolinska. The Pdgfd gene, 100 basepairs from the ATG start codon to the 3'-end of exon 1, was replaced by a lacZ reporter cassette. To identify and track cells that originate from the media we used the validated SMC-specific mouse Cre allele (Myh11CreERT2) combined with the floxed tandem dimer tomato (tdTomato) fluorescent reporter protein gene knocked into the ROSA26 locus (B6.Cg-Gt(ROSA)26Sortm14(CAGtdTomato)Hze/J), in the ApoE null background.

Wild animals

Not used in these studies.

Field-collected samples

None

Ethics oversight

The animal study protocol was approved by the Administrative Panel on Laboratory Animal Care at Stanford University.

Note that full information on the approval of the study protocol must also be provided in the manuscript.

Human research participants

Policy information about studies involving human research participants

Population characteristics

N/A

Recruitment

N/A

Ethics oversight

N/A

Note that full information on the approval of the study protocol must also be provided in the manuscript.

Clinical data

Policy information about clinical studies
All manuscripts should comply with the ICMJEguidelines for publication of clinical research and a completedCONSORT checklist must be included with all submissions.

Clinical trial registration	N/A
Study protocol	N/A
Data collection	N/A
Outcomes	N/A

Dual use research of concern

Policy information about dual use research of concern

Hazards

Could the accidental, deliberate or reckless misuse of agents or technologies generated in the work, or the application of information presented in the manuscript, pose a threat to:

No	Yes	
☒	☐	Public health
☒	☐	National security
☒	☐	Crops and/or livestock
☒	☐	Ecosystems
☒	☐	Any other significant area

Experiments of concern

Does the work involve any of these experiments of concern:

No	Yes	
☒	☐	Demonstrate how to render a vaccine ineffective
☒	☐	Confer resistance to therapeutically useful antibiotics or antiviral agents
☒	☐	Enhance the virulence of a pathogen or render a nonpathogen virulent
☒	☐	Increase transmissibility of a pathogen
☒	☐	Alter the host range of a pathogen
☒	☐	Enable evasion of diagnostic/detection modalities
☒	☐	Enable the weaponization of a biological agent or toxin
☒	☐	Any other potentially harmful combination of experiments and agents

ChIP-seq

Data deposition

☐ Confirm that both raw and final processed data have been deposited in a public database such as GEO.

☐ Confirm that you have deposited or provided access to graph files (e.g. BED files) for the called peaks.

Data access links<br>*May remain private before publication.*	*For "Initial submission" or "Revised version" documents, provide reviewer access links. For your "Final submission" document, provide a link to the deposited data.*
Files in database submission	*Provide a list of all files available in the database submission.*
Genome browser session<br>(e.g. UCSC)	*Provide a link to an anonymized genome browser session for "Initial submission" and "Revised version" documents only, to enable peer review. Write "no longer applicable" for "Final submission" documents.*

Methodology

Replicates	*Describe the experimental replicates, specifying number, type and replicate agreement.*
Sequencing depth	*Describe the sequencing depth for each experiment, providing the total number of reads, uniquely mapped reads, length of reads and whether they were paired- or single-end.*

Antibodies	*Describe the antibodies used for the ChIP-seq experiments; as applicable, provide supplier name, catalog number, clone name, and lot number.*
Peak calling parameters	*Specify the command line program and parameters used for read mapping and peak calling, including the ChIP, control and index files used.*
Data quality	*Describe the methods used to ensure data quality in full detail, including how many peaks are at FDR 5% and above 5-fold enrichment.*
Software	*Describe the software used to collect and analyze the ChIP-seq data. For custom code that has been deposited into a community repository, provide accession details.*

Flow Cytometry

Plots

Confirm that:

- [x] The axis labels state the marker and fluorochrome used (e.g. CD4-FITC).
- [x] The axis scales are clearly visible. Include numbers along axes only for bottom left plot of group (a 'group' is an analysis of identical markers).
- [x] All plots are contour plots with outliers or pseudocolor plots.
- [x] A numerical value for number of cells or percentage (with statistics) is provided.

Methodology

Sample preparation	After 16 weeks of HFD for Pdgfd KO model experiments, or 8 weeks and 16weeks HFD for PDGFD antibody experiments, animals were sacrificed and perfused with phosphate buffered saline (PBS). The aortic root and ascending aorta were excised, up to the level of the brachiocephalic artery, and washed three times in PBS. Collected tissues were placed into an enzymatic dissociation cocktail (2 U /ml liberase, Sigma–Aldrich #5401127001; 2 U/ml elastase, (Worthington, #LS002279) in Hank's Balanced Salt Solution (HBSS)) and minced. After incubation at 37 C for 1 h, the cell suspension was strained, pelleted by centrifugation at 500 x g for 5 min, and resuspended in fresh HBSS. For each scRNA capture, two mice were pooled as a group. Three and two separate pairs of isolation were performed for Ctl and Pdgfd-KO mice, respectively. Two separate pairs of isolations were performed for mice treated with control antibodies (Ctl-ab) or Pdgfd antibodies (PD-ab).
Instrument	Sony SH800
Software	Sony machine LE-SH800 Series software v1.7
Cell population abundance	See plot Suppl. Fig. 3a, tdTomato positive cells were ~45% of the total cells studied.
Gating strategy	Three gates were used 1) FSC-A vs BSC-A was used to gate for the bulk population of cells, 2) FSC-H vs FSC-A was used to minimize doublet sorting, 3) PE-A vs BSC-A was used to determined and gated for comparing to tdTomato positive/negative population of cells.

- [x] Tick this box to confirm that a figure exemplifying the gating strategy is provided in the Supplementary Information.

Magnetic resonance imaging

Experimental design

Design type	*Indicate task or resting state; event-related or block design.*
Design specifications	*Specify the number of blocks, trials or experimental units per session and/or subject, and specify the length of each trial or block (if trials are blocked) and interval between trials.*
Behavioral performance measures	*State number and/or type of variables recorded (e.g. correct button press, response time) and what statistics were used to establish that the subjects were performing the task as expected (e.g. mean, range, and/or standard deviation across subjects).*

Acquisition

Imaging type(s)	*Specify: functional, structural, diffusion, perfusion.*
Field strength	*Specify in Tesla*
Sequence & imaging parameters	*Specify the pulse sequence type (gradient echo, spin echo, etc.), imaging type (EPI, spiral, etc.), field of view, matrix size, slice thickness, orientation and TE/TR/flip angle.*
Area of acquisition	*State whether a whole brain scan was used OR define the area of acquisition, describing how the region was determined.*

Diffusion MRI ☐ Used ☐ Not used

Preprocessing

Preprocessing software	*Provide detail on software version and revision number and on specific parameters (model/functions, brain extraction, segmentation, smoothing kernel size, etc.).*
Normalization	*If data were normalized/standardized, describe the approach(es): specify linear or non-linear and define image types used for transformation OR indicate that data were not normalized and explain rationale for lack of normalization.*
Normalization template	*Describe the template used for normalization/transformation, specifying subject space or group standardized space (e.g. original Talairach, MNI305, ICBM152) OR indicate that the data were not normalized.*
Noise and artifact removal	*Describe your procedure(s) for artifact and structured noise removal, specifying motion parameters, tissue signals and physiological signals (heart rate, respiration).*
Volume censoring	*Define your software and/or method and criteria for volume censoring, and state the extent of such censoring.*

Statistical modeling & inference

Model type and settings	*Specify type (mass univariate, multivariate, RSA, predictive, etc.) and describe essential details of the model at the first and second levels (e.g. fixed, random or mixed effects; drift or auto-correlation).*
Effect(s) tested	*Define precise effect in terms of the task or stimulus conditions instead of psychological concepts and indicate whether ANOVA or factorial designs were used.*

Specify type of analysis: ☐ Whole brain ☐ ROI-based ☐ Both

Statistic type for inference (See Eklund et al. 2016)	*Specify voxel-wise or cluster-wise and report all relevant parameters for cluster-wise methods.*
Correction	*Describe the type of correction and how it is obtained for multiple comparisons (e.g. FWE, FDR, permutation or Monte Carlo).*

Models & analysis

n/a	Involved in the study
☐	☐ Functional and/or effective connectivity
☐	☐ Graph analysis
☐	☐ Multivariate modeling or predictive analysis

Functional and/or effective connectivity	*Report the measures of dependence used and the model details (e.g. Pearson correlation, partial correlation, mutual information).*
Graph analysis	*Report the dependent variable and connectivity measure, specifying weighted graph or binarized graph, subject- or group-level, and the global and/or node summaries used (e.g. clustering coefficient, efficiency, etc.).*
Multivariate modeling and predictive analysis	*Specify independent variables, features extraction and dimension reduction, model, training and evaluation metrics.*
